# Pilot testing a model of psychological care for heart transplant recipients

**DOI:** 10.1186/s12912-016-0183-1

**Published:** 2016-10-26

**Authors:** Aaron Conway, Judith Sheridan, Joanne Maddicks-Law, Paul Fulbrook

**Affiliations:** 1 Institute of Health and Biomedical Innovation, Queensland University of Technology (QUT), 60 Musk Ave, Kelvin Grove, QLD 4059 Australia; 2 School of Psychology, Queensland University of Technology (QUT), Brisbane, Australia; 3 Advanced Heart Failure and Transplant Unit, The Prince Charles Hospital, Chermside, Australia; 4 Nursing Research and Practice Development Centre, The Prince Charles Hospital & School of Nursing, Midwifery and Paramedicine, Australian Catholic University, Sydney, Australia

**Keywords:** Depression, Anxiety, Transplantation, Cognitive behavioural therapy, Psychological, Screening

## Abstract

**Background:**

Anxiety and depression are common after heart transplantation. This study aimed to pilot test the feasibility of a clinical model of psychological care for heart transplant recipients. The model of care involved nurse-led screening for anxiety and depression followed by referral for a course of telephone-delivered cognitive behaviour therapy as well as co-ordination of communication with on-going specialist and primary care services.

**Methods:**

A pilot randomised controlled trial was conducted. Heart transplant recipients who self-reported at least mild anxiety or depressive symptoms were randomised (defined as a score higher than 5 on the Patient Health Questionnaire-9 or the Generalized Anxiety Disorder-7 [GAD-7], or a score higher than 20 on the Kessler Psychological Distress Scale [K10]). The primary outcome was assessment of feasibility of conducting a larger trial, which included identification of recruitment and attrition rates as well as the acceptability of the intervention. Follow-up was conducted at 9 weeks and 6 months.

**Results:**

One hundred twenty-two of the 126 (97 %) heart transplant recipients assessed on their attendance at the outpatient clinic met the study eligibility criteria. Of these patients, 88 (72 %) agreed to participate. A moderate proportion of participants (*n* = 20; 23 %) reported at least mild symptoms of anxiety or depression. Five participants were excluded because they were currently receiving psychological counselling, two withdrew before randomisation and the remaining 13 were randomised (seven to intervention and six to usual care). The majority of the randomised participants were male (*n* = 9; 69 %) and aged over 60 (range 35–73). Median length of time post-transplant was 9.5 years (ranging from 1 to 19 years). On enrolment, 3 randomised participants were taking anti-depressants. One intervention group participant withdrew and a further 3 (50 %) declined the telephone-delivered CBT sessions; all because of restrictions associated with physical illnesses. Attrition was 30 % at the 6 month follow-up time-point.

**Conclusions:**

Due to the poor acceptability of telephone-delivered cognitive behavioural therapy observed in our sample, changes to intervention components are indicated and further pilot testing is required.

**Trial registration:**

ACTRN12613000740796 Date registered: 03/07/2013.

**Electronic supplementary material:**

The online version of this article (doi:10.1186/s12912-016-0183-1) contains supplementary material, which is available to authorized users.

## Background

The International Society for Heart and Lung Transplantation has recommended studies should be conducted to identify interventions that will maximize psychological outcomes after heart transplantation [1]. The society further suggested that intervention studies adapt interventions that have previously demonstrated effectiveness in improving psychological outcomes for patients with other chronic diseases relevant to the heart transplant population. One example of an effective intervention is cognitive behaviour therapy.

Cognitive behaviour therapy (CBT) is a form of psychotherapy, in which therapists help patients overcome psychological symptoms by changing their thinking, behaviour and emotional responses [2]. CBT has proven very effective in the treatment of depression and anxiety, in general, as well as for patients with cardiovascular disease specifically [3]. A recent meta-analysis reported that psychological intervention resulted in moderate improvements in depression and anxiety in patients with coronary heart disease [4]. Also of note, the largest randomised controlled trial of CBT in patients with cardiovascular disease, which randomised 2481 patients post-myocardial infarction, reported that the improvement in psychosocial outcomes at 6 months favoured the group that received counselling (mean [SD] change −10.1 [7.8] vs. -8.4 [7.7]; *p* < 0.01) [5]. This evidence suggests that CBT is likely to decrease anxiety and depression symptoms for heart transplant recipients and therefore should be considered for formal evaluation in a controlled trial.

The ISHLT also recommends: that each heart transplant team should include a psychologist because multidisciplinary treatment teams are better prepared to address psychosocial risk factors for poor outcomes after heart transplantation; that depressive symptoms should be regularly evaluated during follow-up of heart transplant recipients using validated screening instruments; and that all patients who screen positive for symptoms of depression should be referred for specialized treatment [6]. It should be noted, though, that these recommendations are based on consensus. High level evidence demonstrating the benefit of these interventions on heart transplant recipient outcomes is required. For this reason, it was decided to pilot test the feasibility of a model of care that operationalizes these recommendations with the view towards conducting a larger clinical trial. The key features of the model of care that align with the ISHLT recommendations pilot tested in the study reported in this paper include:

- Screening for psychological symptoms;
- Referral for specialized treatment (CBT) for patients with elevated psychological symptoms; and
- Involvement of a multidisciplinary team, including nurses, psychologists, heart transplant physicians and General Practitioner/Primary care providers.

## Methods

A pilot randomised controlled trial was conducted. Human research ethics committee and institutional approval was granted for the study (HREC13QPCH239; 1300000686). It was registered prospectively with the Australian New Zealand Clinical Trials Registry (ACTRN12613000740796).

### Outcomes

The primary outcome of this study was the feasibility of conducting a larger clinical trial. Assessment of feasibility included recruitment issues, likely attrition rates and acceptability of interventions, which are important considerations in planning a larger clinical trial. A planned secondary outcome was to evaluate the potential efficacy of the intervention.

### Participants

Heart transplant recipients aged over 18 years who were scheduled for a routine outpatient consultation with the Heart Transplant Nurse Practitioner at a major metropolitan hospital in Australia were eligible to participate in the study. Patients less than 3 months post-transplant as well as those who were cognitively impaired (as confirmed by a treating clinician), unable to understand and speak English, had a diagnosed major psychiatric comorbidity (schizophrenia, bipolar, dementia) or had a terminal illness (such as cancer) were excluded. Participants who reported at least mild symptoms of anxiety, depression or psychological distress but who were not currently receiving regular psychological therapy from a mental health practitioner were randomised to intervention or control groups.

### Procedure

Prior to their consultation with the Nurse Practitioner, consenting participants completed questionnaires to measure the severity of symptoms commonly associated with anxiety and depression (Patient Health Questionnaire-9 [PHQ-9], Generalized Anxiety Disorder-7 [GAD-7], Kessler Psychological Distress Scale [K10]) as well as questionnaires to evaluate quality of life (Short Form-36 [SF-36]), transplant-specific self-care (Transplant Care Index [TCI], immunosuppression adherence (Basel Assessment of Adherence to Immunosuppressive Medications Scale [BAASIS]) and symptom occurrence and distress (Modified Transplant Symptom Occurrence and Symptom Distress Scale [MTSOSD-R]). The PHQ-9, GAD-7 and K10 results were made available to the Nurse Practitioner during the clinic visit so that usual clinical advice and referrals for patients experiencing psychological distress could be actioned. Participants with scores over 5 on the GAD-7 or PHQ-9 or scores over 20 on the K10 (indicating at least mild anxiety or depressive symptoms) were randomised to the intervention or control group.

### Intervention

All randomised participants received usual care, which involved receiving routine clinical advice from the Heart Transplant Nurse Practitioner for patients identified as experiencing psychological distress as well as a letter to the participant’s General Practitioner advising them of the severity of psychological symptoms and their participation in the study.

Participants randomised to the intervention group were also offered:

- eight telephone-delivered CBT sessions, of about 60 min, delivered by a Doctor of Clinical Psychology student (under the supervision of an accredited Clinical Psychologist); followed by.
- a multidisciplinary case conference between the Heart Transplant Nurse Practitioner, psychologist and the participant’s General Practitioner regarding the implications for on-going psychological care in light of the participant’s response to the course of CBT.

The CBT sessions followed the format recommended by Beck, which involved identifying automatic negative thoughts and practicing reframing techniques, problem-solving, coping strategies and relapse prevention [7]. Participants were encouraged to perform these tasks in the time between their counselling sessions in order to reinforce learnt skills. As medical-surgical recovery was not a focus of the intervention, any questions or concerns related to the clinical aspects of heart transplantation that arose during CBT sessions were to be directed to the Heart Transplant Nurse Practitioner.

Following the course of CBT, the participant’s General Practitioner and members from the Heart Transplant team including the Nurse Practitioner were invited to participate in a case conference. The objective of the case conference was to discuss the patient’s case, their current symptoms, problems they will face and how the problems and symptoms can be managed, with particular attention paid to identifying who will be responsible for particular courses of action. The psychology student who performed the counselling as well as the clinical supervisor for the student were required to attend the conference. Written summaries of the case conferences were to be provided to the participant and each participating health care provider.

### Data collection

Demographics and clinical characteristics, including current medical therapy, were collected from medical records. Psychological symptom experience, quality of life and immunosuppression treatment adherence data were measured using self-report questionnaires. Questionnaires were completed by participants at the outpatient clinic. Follow-up with each randomised participant was conducted by a Research Assistant, who was a Registered Nurse, blinded to treatment allocation at 9 weeks 6 months following baseline assessment. Questionnaires were administered by telephone. At each follow-up data collection point, the research assistant attempted to call the patient over a period of 1 week, with up to four phone calls on different days and at different times of day.

### Measures

#### Patient health questionnaire 9-item scale

The Patient Health Questionnaire 9-item scale (PHQ-9) is a brief self-report measure of depression [8]. Participants were asked to consider the preceding 2 weeks and rate symptom frequency as not at all (0), several days (1), more than half of all days (2) or nearly all days (3). Cut points of 5, 10, and 15 representing mild, moderate, and severe levels of depression have been defined for this instrument [8].

#### Generalized anxiety disorder 7-item scale

The Generalized Anxiety Disorder 7-item scale (GAD-7) is a self-reported measure of anxiety. Higher scores indicate higher levels of anxiety. Participants were asked to consider the preceding 2 weeks and rate symptom frequency as not at all (0), several days (1), more than half of all days (2) or nearly all days (3). A recent validation study of identified that internal consistency was high (α = 0.92) and that it was strongly correlated with the Beck Anxiety Inventory (Spearman’s correlation coefficient = 0.72) [9]. Cut points of 5, 10, and 15 representing mild, moderate, and severe levels of anxiety have been defined for the GAD-7 [9].

#### Kessler psychological distress scale (K-10)

The Kessler Psychological Distress Scale (K-10) measures psychological distress. It is used frequently in research and clinical practice to screen for psychological disorders. The 10 items measure severity of anxiety and depression symptoms using a 5-point Likert type scale. The K-10 strongly discriminates between community cases and non-cases of DSM-IV psychological disorders [10]. A cut-off score of 20 had a sensitivity of 0.66 and specificity of 0.92 for detecting a current anxiety or affective disorder in a population-based survey [11].

#### Medical outcomes short form-36 health survey

The Medical Outcomes Short Form-36 Health Survey (SF-36) yields an 8-scale profile of self-reported functional health and well-being. Higher scores indicate a better health status [12]. The domains of quality of life measured by the SF-36 are physical functioning, role limitations due to physical health, role limitations due to emotional problems, energy/fatigue, emotional well-being, social functioning, pain and general health.

#### Basel assessment of adherence to immunosuppressive medications scale

The Basel Assessment of Adherence to Immunosuppressive Medications Scale (BAASIS) consists of 4 items that measure the ingestion and timing of medications, drug holidays and dose reductions. Non-adherence can be defined as non-adherence on any of the 4 items [13]. The BAASIS also assesses overall adherence using a visual analogue scale (VAS) scale ranging from 0 (never took medications as prescribed) to 100 (always took medications as prescribed). This instrument has sensitivity of 87.5 % and specificity of 78.6 % compared to prospective 1-year virologic failure [14, 15].

#### Transplant care index

The Transplant Care Index (TCI) consists of 6 items rating difficulty in self-care, specifically in relation to transplant-related factors such as attending scheduled health care consultations, following recommended patterns of nutrition and exercise, having scheduled pathology tests, adhering to the prescribed medication regimen and dealing with medication side effects. This instrument demonstrated acceptable internal consistency (Cronbach’s alpha 0.76–0.82) in a sample of 598 renal transplant recipients [16].

#### Modified transplant symptom occurrence and symptom distress-revised

The Modified Transplant Symptom Occurrence and Symptom Distress-Revised (MTSOSD-R) was developed to assess occurrence and distress experienced by the side effects of immunosuppression medications. Content validity, construct validity and discriminant validity of the instrument were substantiated in a study of 108 renal transplant recipients [17, 18]. A subsequent study undertaken with 216 heart transplant recipients demonstrated the internal consistency of this scale. Internal consistency was high for the symptom occurrence (.947) and symptom distress (.984) subscales [19].

### Allocation sequence generation and concealment method

A computer generated table of random sequence was used to randomly allocate participants to one of two conditions (intervention/control). Sealed, sequentially numbered envelopes concealed the randomisation sequence.

### Statistical analysis

Descriptive statistics were used to assess recruitment rates, intervention acceptability and attrition. As per our protocol, it was intended to randomise 30 patients to intervention or control groups in this study because it was expected that this sample would have been sufficient to provide preliminary data to understand the feasibility of the intervention and determine effect size for sample size estimation to adequately power a future larger clinical trial. However, due to the smaller than anticipated number of recruited participants and high proportion of participants randomised to the intervention group not completing the protocol, we could not perform these planned analyses. Descriptive statistics were calculated to describe the characteristics of participants in the study at baseline. Changes in psychological symptom severity from baseline to follow-up were calculated for individual participants but not compared between the intervention and control groups.

## Results

### Recruitment

**Fig. 1:**
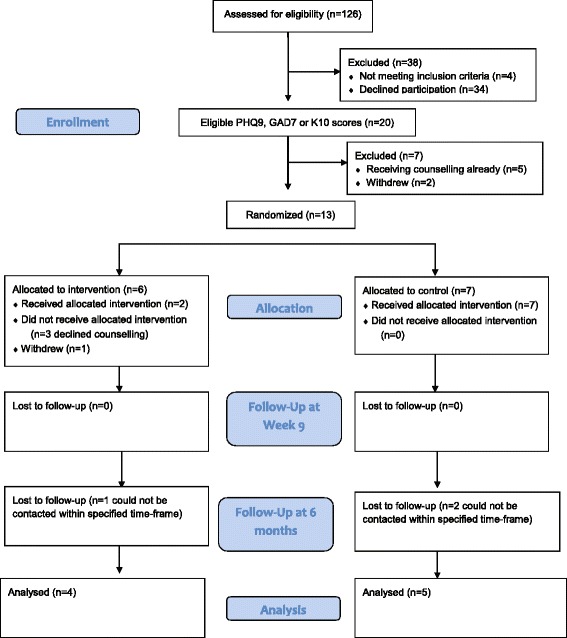
CONSORT Flow diagram

A CONSORT flowchart is presented in Fig. 1. From January to September 2014, 122 of the 126 (97 %) heart transplant recipients assessed on their attendance at the outpatient clinic met the study eligibility criteria. Of these patients, 88 (72 %) agreed to participate. A moderate proportion of participants (*n* = 20; 23 %) reported at least mild symptoms of anxiety or depression. Five of these participants were excluded because they were currently receiving psychological counselling, two withdrew before randomisation and the remaining 13 were randomised.

### Participant characteristics at baseline

An overview of demographic and clinical characteristics of the intervention and control groups is presented in Table 1. The majority of the randomised participants were male (*n* = 9; 69 %) and aged over 60 (range 35–73). Median length of time post-transplant was 9.5 years (ranging from 1 to 19 years). On enrolment, 3 randomised participants were taking anti-depressants. A summary of intervention and control group participants’ health-related quality of life, immunosuppression-related symptom prevalence and treatment adherence is presented in Table 2. Most randomised participants generally did not report difficulties with adherence with immunosuppression therapy (Median score of 98 out of a possible 100 on the BAASIS measure of self-reported overall treatment adherence; Interquartile range 95–100). The majority of the sample reported poor health-related quality of life, with a median score of 50 of a possible 100 for the ‘General Health’ domain of the SF-36 (IQR 50–55).

**Table 1:** Baseline demographic and clinical characteristics

Variable	Intervention *n* = 6	Control *n* = 7
	Median (range) or Count (%)	Median (range) or Count (%)
Age	57 (43–70)	61 (35–73)
Male	4 (67)	5 (71)
Years since transplant	9 (2–19)	10 (1–18)
Number of comorbidities	8 (3–8)	8 (7–10)
History of anxiety or depression disorder	3 (50)	1 (14)
Taking anti-depressants	1 (14)	2 (29)

**Table 2:** Baseline severity of psychological and immunosuppression-related symptoms and levels of health-related quality and treatment adherence

Variable	Intervention *n* = 6 Median (IQR)	Control *n* = 7 Median (IQR)
PHQ-9 score	9 (7–13)	10 (8–18)
GAD-7 score	4.5 (2–7)	8 (7–10)
K-10 score	19.5 (17–21)	20 (16–25)
Difficulty with keeping scheduled follow-up visits^d^	5 (4–5)	4 (4–5)
Difficulty with following a regular exercise program^d^	2.5 (1–4)	2 (1–3)
Difficulty with following a healthy and balanced diet^d^	4 (2–4)	2 (2–3)
Difficulty with having tests done as scheduled^d^	5 (5–5)	4 (4–5)
Difficulty with taking all medicines as prescribed^d^	5 (5–5)	4 (4–5)
Difficulty with side effects of medicines^d^	4.5 (2–5)	4 (3–4)
Immunosuppression treatment adherence^a^	100 (100–100)	95 (90–98)
Number of immunosuppression-related symptoms experienced ‘almost always’ or ‘always’^b^	6.5 (3–12)	7 (3–12)
Physical functioning^c^	70 (25–95)	40 (15–55)
Role limitations due to physical health^c^	50 (0–75)	25 (0–75)
Role limitations due to emotional problems^c^	0 (0–100)	100 (0–100)
Energy/fatigue^c^	27.5 (5–50)	30 (20–35)
Emotional well-being^c^	70 (64–72)	60 (60–64)
Social functioning^c^	50 (50–63)	50 (50–50)
Pain^c^	36 (33–48)	50 (35–58)
General health^c^	53 (50–55)	50 (40–50)

*IQR* interquartile range, ^a^Rating of overall treatment adherence over the past 2 weeks on a scale of 0–100 (higher score = better adherence); ^b^MTSOSD-59; ^c^Scores range from 0 to 100 with higher scores representing better health-related quality of life’; ^d^1 = Very difficult, 5 = Very easy; PHQ-9 = Patient Health Questionnaire 9-item scale; GAD-7 = Generalized Anxiety Disorder 7-item scale; K-10 = Kessler Psychological Distress Scale

### Acceptability of intervention components

After randomisation, one participant from the intervention group withdrew from the study. A further 3 (50 %) of the intervention participants declined the telephone-delivered CBT sessions; all because of restrictions associated with physical illness. Two intervention participants (33 %) completed the CBT sessions. Neither of these participants had a history of a diagnosed anxiety or depression disorder. A case conference with one of the intervention participant’s general practitioners could not be scheduled at a time that suited all members of the transplant team. The other intervention participant who completed the course of CBT requested that the case conference not be conducted. Written reports from the psychologist were circulated instead.

### Attrition

All participants who had not withdrawn from the study completed the follow-up questionnaires at 9 weeks after screening. One participant (16 %) in the intervention group and two participants (29 %) in the control group were not able to be contacted for 6 month follow-up.

### Changes in severity of psychological symptoms

Psychological symptoms measured with the PHQ-9 and GAD-7of the two participants (33 %) who completed the course of CBT reduced to scores below 5 at follow-up conducted at 9 weeks post-screening. All intervention group participants who declined CBT (*n* = 3; 67 %) still reported psychological symptoms with a score greater than 5 on the PHQ-9 or GAD-7 at the 9 week follow-up (*n* = 1 withdrew after randomisation). Four (57 %) of the seven control group participants still experienced symptoms with a score greater than 5 on the PHQ-9 or GAD-7 at the 9 week follow-up. At 6 month follow-up, two participants (40 %) in the control group reported psychological symptoms with a score greater than 5 on the PHQ-9 or GAD-7 (two participants were lost to follow-up). All participants in the intervention group who completed the follow-up questionnaire at 6 months after screening reported PHQ-9 and GAD-7 scores <5 (*n* = 4; 1 participant had withdrawn and one participant could not be contacted).

## Discussion

The main finding of this pilot study is that the majority of heart transplant recipients who reported at least mild symptoms of anxiety or depression on screening at their routine outpatient appointment but were not currently receiving psychological counselling did not accept a telephone-delivered CBT intervention. Results may reflect the broader difficulties in treating mental health problems stemming from perceived risk of stigmatisation associated with being diagnosed with a psychological disorder [20]. However, participants’ decisions to accept psychological treatment may also have been influenced by the unique experiences associated with heart transplantation. Qualitative studies have identified that competing senses of hope and gratitude mixed with guilt and grief regarding the acceptance of a heart from a deceased donor may contribute to anxiety and depression in this population [21]. In this regard, a noteworthy finding is that the acceptability of telephone-delivered CBT was worse in our sample of heart transplant recipients compared with other chronic disease populations. For example, the MoodCare study similarly involved a course of telephone-delivered CBT for patients hospitalised with acute coronary syndrome who reported depressive symptoms with a PHQ-9 score more than 5 [22]. Yet in the Mood Care study, 61 % of the 61 participants who were randomised to the intervention group completed five or more CBT sessions and the median number of sessions was eight [22]. It should be noted, though, that 32 % of participants in the Mood Care study who were eligible for inclusion based on initial screening that took place in hospital either withdrew or were lost to follow-up at a second screening undertaken just prior to randomisation [22]. Future trials involving psychological interventions for heart transplant recipients should therefore consider utilising a two-stage screening process to confirm participants’ intentions in taking part in CBT before randomisation.

Due to the poor acceptability of telephone-delivered CBT observed in our sample, changes to intervention components are indicated and further pilot testing should be conducted before proceeding to a clinical trial of a psychological intervention for heart transplant recipients with anxiety or depression. One change to consider piloting would be to raise the screening cut-off scores. Alongside this pilot study, we evaluated the validity and reliability of the PHQ-9, GAD-7 and K-10 for detection of anxiety or depression. Full details are reported elsewhere (under review). It was identified that the optimal cut-off score for detecting depression was a score of 10, which is considerably higher than the score we used for eligibility to be randomised to CBT in the pilot study. Moreover, it is known that PHQ-9 scores may include a small amount of variance from somatic symptoms not related to depression [23]. For these reasons, it is not surprising that some intervention group participants declined CBT due to physical illness. These participants may have accumulated a PHQ-9 score >5 due to the severity of somatic symptoms contained within the PHQ-9 that were not necessarily caused by depression, such as fatigue and sleep disturbance. An alternative to raising the cut-off score in an effort to decrease false-positive screening is to conduct a structured psychological interview with heart transplant recipients who screen positive for a potential psychological disorder. Psychological interventions can then be targeted at only those heart transplant recipients who meet criteria for a psychological disorder.

A further alternative, which would be prudent to investigate based on results from this pilot study that indicated a 2 month period of counselling was not acceptable to most participants, is to consider the use of nurse-led brief interventions to improve psychological outcomes for heart transplant recipients. A nurse-led brief CBT intervention has been shown to be accepted well by hospitalised heart failure patients. In a randomised controlled trial, hospitalised heart failure patients who reported mild-moderate symptoms of depression as indicated by Beck Depression Inventory scores of 10–28 were randomised to usual care or a single, nurse-delivered, 30-min, one-on-one cognitive therapy session that took place in a hospital followed by a 5–10 min telephone ‘booster’ that was conducted 1 week after discharge [24]. In this study, 20 of the 21 patients randomised to the intervention group received the allocated intervention [24].

It was planned to investigate the potential efficacy of the intervention on health-related quality of life, severity and distress of immunosuppression-related symptoms and treatment adherence by calculating effect sizes. However, this was not conducted due to the smaller than anticipated sample size. Reassuringly, both participants who received CBT as part of the study had remission of their symptoms after the end of the 8-week course of counselling. This is consistent with the previous literature in other populations that has overwhelmingly demonstrated that CBT reduces the severity of psychological symptoms and that telephone-delivered CBT is equally as effective as a face to face counselling strategy [25, 26].

A limitation of this pilot study to consider is that it was conducted at only one transplantation centre. Also, selection bias might have influenced the results. Heart transplant recipients suffering from anxiety or depression may have been less inclined to participate in the research [27]. Heart transplant recipients were also screened for the presence of anxiety or depression only once during the data collection period, even if they attended the outpatient clinic multiple times over that period. Potentially, screening at each attendance would have increased detection of anxiety or depression.

## Conclusions

Results from our study indicated that many heart transplant recipients who might have benefited from psychological intervention did not accept treatment in the form of telephone-delivered CBT. It is unknown whether or not other modes of delivering CBT (face to face or online) would be more or less acceptable. These are potential areas to consider pursuing in further research aimed at improving psychological outcomes for heart transplant recipients. Due to the poor acceptability of telephone-delivered CBT observed in our sample, changes to intervention components are indicated and further pilot testing should be conducted before proceeding to a large clinical trial of a psychological intervention for heart transplant recipients with anxiety or depression.

## Additional files

Additional file 1: Dataset. (XLS 27 kb)

## Abbreviations

BAASIS: Basel assessment of adherence to immunosuppressive medications scale
CBT: Cognitive behavioural therapy
GAD-7: Generalized anxiety disorder-7
IQR: Interquartile range
ISHLT: International society for heart and lung transplantation
K10: Kessler psychological distress scale
MTSOSD-R: Modified transplant symptom occurrence and symptom distress scale
PHQ-9: Patient health questionnaire-9
SF-36: Medical outcomes short form health survey-36
TCI: Transplant care index
